# Assessment of effectiveness of optimum physical distancing phenomena for COVID-19

**DOI:** 10.1063/5.0046429

**Published:** 2021-05-10

**Authors:** Branson Chea, Andre Bolt, Martin Agelin-Chaab, Ibrahim Dincer

**Affiliations:** Faculty of Engineering and Applied Science, Ontario Tech University, 2000 Simcoe Street North, Oshawa, Ontario L1H 7K4, Canada

## Abstract

Currently, COVID-19 is a global pandemic that scientists and engineers around the world are aiming to understand further through rigorous testing and observation. This paper aims to provide safe distance recommendations among individuals and minimize the spread of COVID-19, as well as examine the efficacy of face coverings as a tool to slow the spread of respiratory droplets. These studies are conducted using computational fluid dynamics analyses, where the infected person breathes, coughs, and sneezes at various distances and environmental wind conditions and while wearing a face-covering (mask or face shield). In cases where there were no wind conditions, the breathing and coughing simulations display 1–2 m physical distancing to be effective. However, when sneezing was introduced, the physical distancing recommendation of 2 m was deemed not effective; instead, a distance of 2.8 m and greater was found to be more effective in reducing the exposure to respiratory droplets. The evaluation of environmental wind conditions necessitated an increase in physical distancing measures in all cases. The case where breathing was measured with a gentle breeze resulted in a physical distancing recommendation of 1.1 m, while coughing caused a change from the previous recommendation of 2 m to a distance of 4.5 m or greater. Sneezing in the presence of a gentle breeze was deemed to be the most impactful, with a recommendation for physical distancing of 5.8 m or more. It was determined that face coverings can potentially provide protection to an uninfected person in static air conditions. However, the uninfected person's protection can be compromised even in gentle wind conditions.

## I. INTRODUCTION

Throughout history, mankind has always been faced with plagues and illnesses that have threatened the well-being of society.^1^ It has always been the aim of scientists, engineers, and researchers to work toward creating innovative solutions to overcome diseases and other issues.^2^ Most recently, the world is in the midst of combating COVID-19, a global outbreak.^3^ COVID-19 is a zoonotic disease caused by a new strain of coronavirus that began in December 2019. Symptoms from the virus include fever, shortness of breath, nausea, congestion, and a multitude of others.^4^ Similar to other pathogenic respiratory coronaviruses such as severe acute respiratory syndrome (SARS) and the Middle East respiratory syndrome (MERS), the effects of COVID-19 can be fatal, given its 3% mortality rate.^5^ The virus can also cause severe, long-lasting health complications such as inflammation of the heart, referred to as myocarditis and pericarditis. While cases of myocarditis are uncommon among young people, it can still occur.^6^ Presently, cases of the virus have been reported in more than 200 countries, with outbreaks occurring in hospitals, old age care facilities, prisons, and hospitals.^7^

During the preliminary stages of COVID-19 research, it was believed that human to human transmissions of the virus was relatively limited and posed no imminent threat. However, as further research on the virus continued, it was established that the virus was being transmitted from human to human.^5^ As recent as April 2020, scientists were able to determine that the primary mode of transmission of the virus is attributed to respiratory droplets when the unprotected individuals are in close proximity with an infected person.^8–10^ Additional evidence suggests that the virus can be found in blood and human stool and can exist on surfaces.^8^

In order to stop and mitigate the transmission of COVID-19, health care professionals recommend practicing hand hygiene, maintaining physical distancing, wearing masks and face coverings, and using cleaning or disinfectant supplies.^11^ Of the methods listed, wearing a mask and other face coverings is the most logical method to stop the transmission of the virus through respiratory droplets.^12^ In this race to understand the spread of the virus better, studies have been completed to examine the spread of the virus through common modes of transmission, such as coughing and sneezing. The impact of wearing face coverings and masks was analyzed by numerous researchers. This review provides information on how COVID-19 and similar viruses are transmitted through fluids.

In a study conducted by Bourouiba,^13^ the impact that respiratory emissions have on the transmission of COVID-19 was examined. Based on the analysis conducted, respiratory emission consisted of exhalations, coughs, and sneezes. The fluid flow from the transmission can be described as a multiphase turbulent gas cloud containing a cluster of mucosalivary droplets. Bourouiba^13^ determined that droplets can travel 7–8 m. However, the Center for Disease Control and Prevention (CDC) recommends approximately 2 m of distance to mitigate spread. Also, the scientific advisory group of the United Kingdom estimated that the risk of COVID-19 transmission is 2–10 times greater at 1 m of physical distance as opposed to 2 m.^14^ According to Jones *et al.*,^14^ the origins of the 2 m of physical distancing rule date as far back as 1897 where the researcher, Flugge, suggested that 1–2 m be an adequate amount of physical distance that visible droplets containing pathogens were able to travel. Additionally, in 1948 a study was conducted to examine the spread of hemolytic streptococci, where 65% of participants produced large respiratory droplets, of which less than 10% produced large respiratory droplets that were able to travel up to 1.7 m. Jones *et al.*^14^ also referred to a study conducted in 1934 by Wells^15^ where large respiratory droplets were compared to small respiratory droplets. It was identified that larger respiratory droplets fell between a distance of 1–2 m before they were able to evaporate. In contrast, smaller respiratory droplets (aerosols) evaporated before they reached the ground.

The concept of 2 m of physical distancing , which was technically found to be inadequate, was further explained by Jones *et al.*^14^ by outlining that airflow velocity is an important characteristic that must be taken into consideration. By not considering airflow velocity, smaller particles will encounter more drag; hence, they will land closer to the person releasing the respiratory droplet, whereas the larger droplet can potentially travel further and land within the 1–2 m range. However, if exhaled airflow is taken into consideration, respiratory droplet clouds can travel much further distances, exceeding 2 m. Setti *et al.*^16^ also outline why 2 m of physical distancing may not be adequate; however, they acknowledged that it can be considered a reasonable protective measure if people involved wear a mask during daily activities.

In another study, Busco *et al.*^17^ analyzed the impact sneezing has on the spread of the virus. The experimental model consisted of a 100 ml buffer chamber, a pressure outlet, a micro-dynamic pressure transducer, a power supply, and a data acquisition system. In the experiment, the subject sneezes, then the pressure the sneeze is released at is recorded between 0 and 0.5 s. It was found that the pressure the sneeze was released at peaked at about 0.1 s with a pressure between 8000 and 9000 Pa. In the theoretical model, Busco *et al.*^17^ used computational fluid dynamics (CFD) to simulate the distance a sneeze can travel. The sneeze had a starting velocity of 17 m/s, a spread angle of 25°, and the angle between the sneezing axis and the horizontal direction was −27.5°. The flow pattern for the experimental and theoretical model closely resembled each other; however, the results for the experimental model were not recorded past 1.5 m. The theoretical model was able to show how far the sneeze was able to travel, depending on how the subject's head was tilted. The study found that when the subject sneezes without any tilt in their neck, the sneeze was able to travel up to 4 m, with the particles with a larger droplet diameter (>500 *μ*m) being concentrated 2 to 4 m. It was also established that in this instance, the majority of the sneeze was contained within 2 m. A similar study was also conducted by Cummins *et al.*,^18^ where the trajectory of respiratory particles was analyzed in the x and y-direction.

In order to determine the impact ventilation has on the spread of COVID-19, Bhagat *et al.*^19^ conducted a comprehensive fluid flow study, comprising of a series of simulations and a brief experimental portion. Bhagat *et al.*^19^ used differential synthetic Schlieren images to observe fluid flow within the environment. The study primarily examined the equations required to form the ventilation system used to remove the subject's exhalation in the room. Since CO_2_ is released during exhalation, the concentration of CO_2_ within the environment was used to indicate the likely hood of the virus being present within the environment. Gao *et al.*^20^ also conducted a CFD simulation due to the SARS epidemic. The simulation examined the spread of respiratory droplets within a ventilated room. In the simulation, both the polluting and exposed person were sitting in the center of a room of dimensions 2.2 m by 2.6 m. The ventilated air was provided at the bottom side of the room at floor level, with an inlet velocity and temperature of 0.2 m/s and 22 °C. The outlet vent of the room was located along the top of the opposite wall. The air within the room was ventilated at 0.024 m^3^/s, enabling the room to filter out old air at 0.024 m^3^/s. Unlike other studies that only examined coughing and sneezing, this study also examined regular inhalation and exhalation from within a ventilated room, where the subjects are close to each other (1.2 m). The velocity path lines during inhalation and exhalation from the mouth and nose greatly resemble each other; however, more air particles are transmitted from the mouth as opposed to the nose. During inhalation, air flows in a stream under the person's mouth and then moves upward toward the exit vent during exhalation. It was noted that the path lines did not come in contact with the exposed person during this simulation. This indicated an exposed person is at significantly less risk of exposure from the infected person in a room that is ventilated, as opposed to not ventilated, given the specifications provided within the simulation. However, Gao *et al.* also found that the ventilation provided within the room would not be adequate enough to protect the exposed person if the polluting person sneezed. They stated that 1 s was an adequate amount of time to reach the exposed person's area of breathing. Yu *et al.*^21^ also conducted a CFD simulation due to the SARS epidemic; however, they analyzed its spread within an apartment building rather than confined to a room. Vuorinen *et al.*^22^ also conducted a similar study to investigate the transmission of COVID-19 indoors. The respiratory fluid droplet size and flow within the environment were considered in these studies. Unlike the study conducted by Gao *et al.*, which used an office setting for the simulation, Vuorinen *et al.*^22^ conducted a simulation within a retail store between aisles, with a mixing fan and exhaust vent located above. Vuorinen *et al.*^22^ used the Navier–Stokes equations for the evolution of a cough and the velocity field.

Airborne transmission of COVID-19 was also investigated by Feng *et al.*^23^ through a CFD analysis. This study aimed to evaluate how wind and relative humidity impact the effectiveness of physical distancing in mitigating the spread of COVID-19. In the given simulation, human models were positioned to face each other 1.83 m (6 ft) away and 3.05 m (10 ft) away in different simulations. In regard to the environment, a light breeze (1.0 m/s), gentle breeze (3.9 m/s), and a moderate breeze (5.5 m/s) were considered in simulations. The relative humidity within the environment considered was 99.5% and 40%. The study also measured the potential for masks to mitigate the risk of exposure to COVID-19, with a large emphasis placed on N95 masks. The study established that if an infected person coughs in a static air environment or a dynamic air environment where airflow in the direction of the cough, micron-size respiratory droplets pose a risk to people 6 feet and 10 feet away in many instances. It was also identified that a relative humidity of 40% within the environment can cause the respiratory droplets to evaporate and spend a prolonged period in the air. Furthermore, Feng *et al.*^23^ also placed the simulated mask 1.8 cm away from the person's face to replicate improper fitting of the mask. The study established that even by wearing the mask in such an unrealistic condition, the ability to transmit droplets when coughing is drastically minimized. Another element studied was the ease of transmission from respiratory droplets when face coverings were used, such as face shields and masks. However, it should be noted that the study considered the filtration efficiency of different types of masks, and N95/K95 did perform the best. Pendar *et al.*^24^ also conducted a very similar study, where the model displayed the interaction respiratory particles have with their environment after their release. In this study, the velocity, angles of release, and surface area the particles were released at were varied within the simulation. The simulations were very comprehensive; it recorded the average diameter of the saliva and the length/width traveled. The study also observed the impact of wearing a face covering on the spread of respiratory droplets. It was established that when the sneeze was released at an initial velocity of 22.3 m/s, average size particles (90 *μ*m) traveled approximately 2.3 m, whereas larger droplets with a diameter of 540 *μ*m traveled over 4 m. Diwan *et al.*^25^ also conducted another numerical simulation where cough and sneeze flow was analyzed. The distance traveled in length and width was recorded; the study established a larger emphasis on the environmental temperature and the impact it has on the dispersion of respiratory droplets.

Leonard *et al.*^26^ examined how the spread of COVID-19 can be mitigated through nasal inhalation with a surgical mask. The use of surgical masks has been recommended by numerous health care professionals to slow down the spread of the virus.^27^ Leonard *et al.*^26^ further established the importance of wearing a mask using a CFD analysis. In their study, Leonard *et al.*^26^ determined that 88.8% of the particulate mass was able to be captured with a surgical mask. The study highlighted that the majority of the respiratory fluids that did escape were attributed to poor-fit and mask design. External environmental conditions and physical distancing were not made a primary focus of the study.

In this paper, CFD analyses are conducted to evaluate how respiratory droplets are transmitted and to measure the effectiveness of face coverings. As previously stated, the virus is primarily transmitted through respiratory droplets. Therefore, the proposed simulations consider sneezing, coughing, and breathing as the primary modes of releasing respiratory droplets. Each simulation consists of two human models, one infected person and the other uninfected. A variety of environmental conditions such as wind speed and wind direction are also considered to create variance within the study and account for unpredictability within the environment. To measure the effectiveness of face coverings, simulations are conducted in which the infected subject was not wearing any form of face coverings as a reference when measuring the distance to which respiratory particles reached. Based on the results obtained from the various simulations, recommendations can be made.

## II. METHODOLOGY

The methodology will first discuss the geometry and computational domain created for the simulation. The numerical approach for flow modeling, turbulence, the multiphase and discrete phase models, and porous conditions will follow. Finally, the mesh generation techniques and solver settings are listed.

### A. Geometry and Domain

**FIG. 1.**
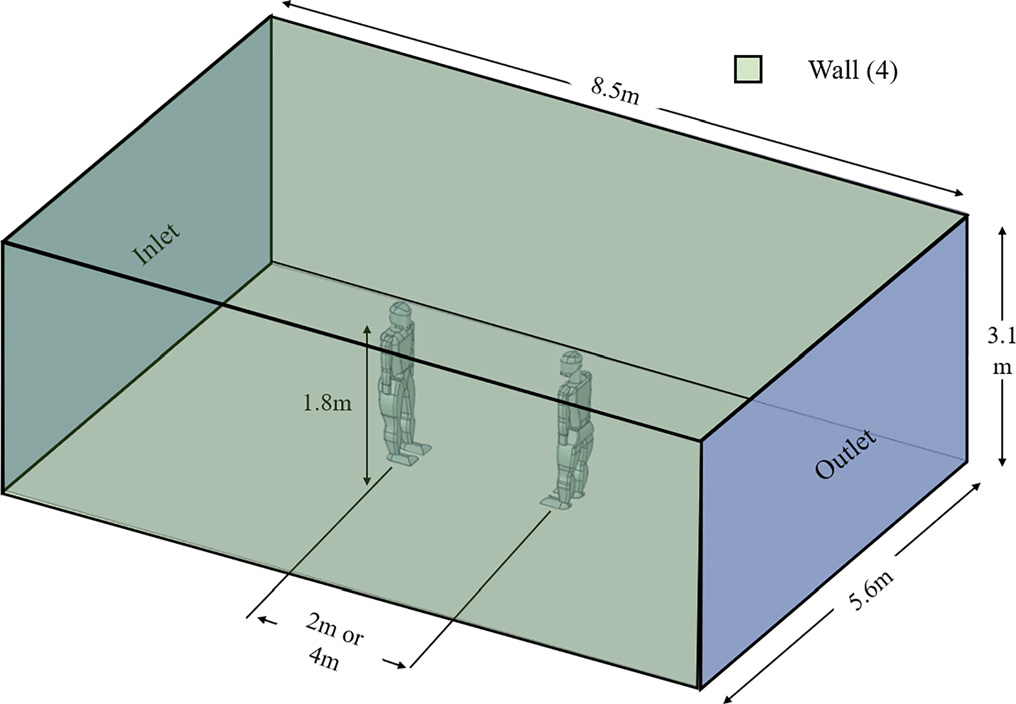
Domain generated for the study.

**FIG. 2.**
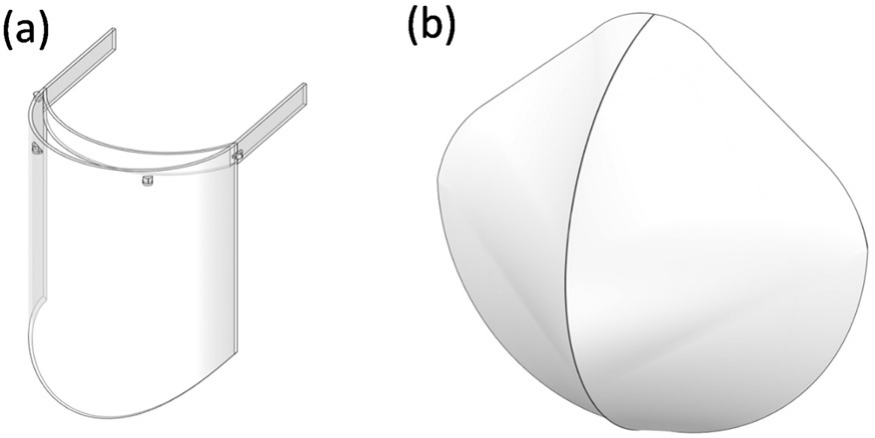
Models of (a) face shield and (b) N95 mask.

The study features several models developed for the study. The computational domain generated for the study is pictured in Fig. 1. The computational domain was designed to mimic outdoor conditions. In Fig. 1, the distance between the human models varies between experiments with 2 m or 4 m. The lateral, upper, and lower boundaries are specified as wall conditions in the analysis, as indicated in Fig. 1. Additionally, the face shield and N95 mask models were developed for the study based on standard dimensions available in the market, as shown in Fig. 2.

### B. Numerical Approach

The numerical approach is broken down into several aspects. First, flow modeling is introduced with continuity, momentum, and turbulence sources discussed. Second, the discrete phase model and multiphase model introduces the particle droplets injection into the simulation. The final aspect discussed is the porous zone, which is introduced for the N95 mask used in the simulations.

#### 1. Flow modeling

The general form for flow modeling is valid for both incompressible and compressible flow. The continuity equation is expressed as follows:^28^

$$\frac{\partial \rho }{\partial t}+\nabla \cdot \left(\rho \vec{v}\right)=S_{\mathrm{DPM}}+S_{\mathrm{other}}.$$ (1)

The momentum conservation equation used for a non-accelerating reference frame^28^ is represented as follows:

$$\frac{\partial }{\partial t}\left(\rho \vec{v}\right)+\nabla \cdot \left(\rho |\vec{v}|\vec{v}\right)=-\nabla p+\nabla \cdot \overset{̿}{\tau }+\rho \vec{g}+{\vec{F}}_{\mathrm{DPM}}+{\vec{F}}_{\mathrm{other}}.$$ (2)

The stress tensor is represented as follows,^28^ where the molecular viscosity is represented by μ and *I* is the unit tensor:

$$\overset{̿}{\tau }=\mu \left[\left(\nabla \vec{v}+\nabla {\vec{v}}^{T}\right)-\frac{2}{3}\nabla \cdot \vec{v}I\right].$$ (3)

The simulation features two phases; thus, a momentum equation for phase q is represented as follows:^28^

$$\frac{\partial }{\partial t}\left(\alpha_{q}\rho_{q}{\vec{v}}_{q}\right)+\nabla \cdot \left(\alpha_{q}\rho_{q}{\vec{v}}_{q}\right)=-\alpha_{q}\nabla p+\nabla \cdot {\overset{̿}{\tau }}_{q}+\alpha_{q}\rho_{q}\vec{g}+\sum_{p=1}^{n}\left({\vec{R}}_{pq}+{\dot{m}}_{pq}{\vec{v}}_{pq}-{\dot{m}}_{qp}{\vec{v}}_{qp}\right)+({\vec{F}}_{q}+{\vec{F}}_{\mathrm{lift},q}+{\vec{F}}_{wl,q}+{\vec{F}}_{vm,q}+{\vec{F}}_{td,q}).$$ (4)

The phase stress–strain tensor, ${\overset{̿}{\tau }}_{q}$, can be calculated as follows:^28^

$${\overset{̿}{\tau }}_{q}=\alpha_{q}\mu_{q}\left(\nabla {\vec{v}}_{q}+{\vec{v}}_{q}^{T}\right)+\alpha_{q}\left(\lambda_{q}-\frac{2}{3}\mu_{q}\right)\nabla \cdot {\vec{v}}_{q}\overset{̿}{I}.$$ (5)

${\vec{F}}_{q}$, ${\vec{F}}_{\mathrm{lift},q}$, ${\vec{F}}_{wl,q}$, ${\vec{F}}_{vm,q}$, and ${\vec{F}}_{td,q}$ represent the external body, lift, wall lubrication, virtual mass, and turbulent dispersion forces, respectively. The interaction force between phases is ${\vec{R}}_{pq}$. The shear and bulk velocity for phase q are represented by $\mu_{q}$ and $\lambda_{q}$, respectively.

#### 2. Turbulence

The turbulence model selected for use in this paper is the k–ω shear stress transport (SST) model, a refinement to the k–ω and k–ϵ model, resulting in a more accurate and reliable model.^28^ The k–ω SST model was introduced by Menter,^29^ where the use of a blending function aids in switching between k–ω for freestream flows and k–ϵ for near wall flow.

The transport equation for the k–ϵ is expressed as follows:^28^

$$\frac{\partial }{\partial t}\left(\rho k\right)+\frac{\partial }{\partial x_{i}}\left(\rho ku_{i}\right)=\frac{\partial }{\partial x_{j}}\left(\Gamma_{k}\frac{\partial k}{\partial x_{j}}\right)+G_{k}-Y_{k}+S_{k}+G_{b}.$$ (6)

The transport equation for the k–ω is expressed as follows:^28^

$$\frac{\partial }{\partial t}\left(\rho \omega \right)+\frac{\partial }{\partial x_{i}}\left(\rho \omega u_{i}\right)=\frac{\partial }{\partial x_{j}}\left(\Gamma_{\omega }\frac{\partial \omega }{\partial x_{j}}\right)+G_{\omega }-Y_{\omega }+S_{\omega }+G_{\omega b},$$ (7)

where $G_{k}$ and $G_{\omega }$ represent the production of turbulent kinetic energy and ω, respectively. Additionally, $Y_{k}$ and $Y_{\omega }$ represent the dissipation of turbulent kinetic energy and ω, respectively.

The effective diffusivities ($\Gamma_{k}$ and $\Gamma_{\omega }$) from the transport equations are as follows:^28^

$$\Gamma_{k}=\mu +\frac{\mu_{t}}{\sigma_{k}},$$ (8)

$$\Gamma_{\omega }=\mu +\frac{\mu_{t}}{\sigma_{\omega }}.$$ (9)

The turbulent Prandtl numbers for k ($\sigma_{k}$) and ω ($\sigma_{\omega }$) are calculated as follows:^28^

$$\sigma_{k}=\frac{1}{\frac{F_{1}}{\sigma_{k,1}}+\frac{1+F_{1}}{\sigma_{k,2}}},$$ (10)

$$\sigma_{\omega }=\frac{1}{\frac{F_{1}}{\sigma_{\omega ,1}}+\frac{1+F_{1}}{\sigma_{\omega ,2}}}.$$ (11)

Here, F_1_, $\phi_{1}$, and $D_{\omega }^{+}$ can be calculated as follows:

$$F_{1}=\tanh\left(\phi_{1}^{4}\right),$$ (12)

$$\phi_{1}=\text{min}\left[\text{max}\left(\frac{\sqrt{k}}{0.09\omega y},\frac{500\mu }{\rho y^{2}\omega }\right),\frac{4\rho k}{\sigma_{\omega ,2}D_{\omega }^{+}y^{2}}\right],$$ (13)

$$D_{\omega }^{+}=\text{max}\left[2\rho \frac{1}{\sigma_{\omega ,2}}\frac{1}{\omega }\frac{\partial k}{\partial x_{j}}\frac{\partial \omega }{\partial x_{j}},10^{-10}\right].$$ (14)

The turbulent viscosity ($\mu_{T}$) is unique for the k–ω SST model with S characterizing the strain rate magnitude and $\alpha^{*}$ representing the turbulent viscosity damping coefficient. $\mu_{T},F_{2}$, $\mathrm{and}\phi_{2}$ can be calculated as follows:^28^

$$\mu_{t}=\frac{\rho k}{\omega }\frac{1}{\text{max}\left[\frac{1}{\alpha^{*}},\frac{SF_{2}}{a_{1}\omega }\right]},$$ (15)

$$F_{2}=\tanh\left(\phi_{2}^{2}\right),$$ (16)

$$\phi_{2}=\text{max}\left[2\frac{\sqrt{k}}{0.09\omega y},\frac{500\mu }{\rho y^{2}\omega }\right],$$ (17)

#### 3. Dense discrete phase model

The use of the dense discrete phase model is used to model the particle ejection via sneezing, coughing, and breathing into the domain by the human model. The mass conservation equation for the particulate phase is written as follows:^28^

$$\frac{\partial }{\partial t}\left(\alpha_{p}\rho_{p}\right)+\nabla \cdot \left(\alpha_{p}\rho_{p}{\vec{v}}_{p}\right)=\sum_{q=1}^{\mathrm{nphase}}\left({\dot{m}}_{pq}-{\dot{m}}_{qp}\right).$$ (18)

The momentum conservation equation for the particulate phase is represented as follows:^28^

$$\frac{\partial }{\partial t}\left(\alpha_{p}\rho_{p}{\vec{v}}_{p}\right)+\nabla \cdot \left(\alpha_{p}\rho_{p}{\vec{v}}_{p}{\vec{v}}_{p}\right)=-\alpha_{p}\nabla \;p+\nabla \cdot \left[\alpha_{p}\mu_{p}\left(\nabla {\vec{v}}_{p}+{\vec{v}}_{p}^{T}\right)\right]+\alpha_{p}p_{p}\vec{g}+F_{vm,\mathrm{lift},\mathrm{user}}+\sum_{q=1}^{\mathrm{nphase}}\left({\vec{K}}_{qp}\left({\vec{v}}_{q}-{\vec{v}}_{p}\right)+{\dot{m}}_{qp}{\vec{v}}_{qp}-{\dot{m}}_{qp}{\vec{v}}_{qp}\right)+K_{\mathrm{DPM}}\left({\vec{v}}_{\mathrm{DPM}}-{\vec{v}}_{p}\right)+S_{\mathrm{DPM},\mathrm{explicit}}.$$ (19)

#### 4. Eulerian multiphase model

The multiphase turbulent dispersion forces for the fluid particles are examined using the Eulerian model. Turbulent drag is modeled as follows:

$$K_{pq}\left({\vec{\tilde{v}}}_{p}-{\vec{\tilde{v}}}_{q}\right)=K_{pq}\left({\vec{v}}_{p}-{\vec{v}}_{q}\right)-K_{pq}{\vec{v}}_{dr}.$$ (20)

Here, instantaneous drag is represented as the left term, K_pq_ represents an interphase exchange coefficient. The turbulent dispersion force is characterized by $K_{pq}{\vec{v}}_{dr}$.

#### 5. Porous zone

The inclusion of porous media in the simulations is to model the effect of the N95 mask. In order to include the effect, a momentum source term (S_i_) is applied to the fluid flow equations. The magnitude of velocity is described as $\left|v\right|$ and matrices are represented by D and C. The equation is expressed as follows:^28^

$$S_{i}=-\left(\sum_{j=1}^{3}D_{ij}\mu v_{j}+\sum_{j=1}^{3}C_{ij}\frac{1}{2}\rho \left|v\right|v_{j}\right).$$ (21)

### C. Mesh generation

#### 1. Mesh details

**FIG. 3.**
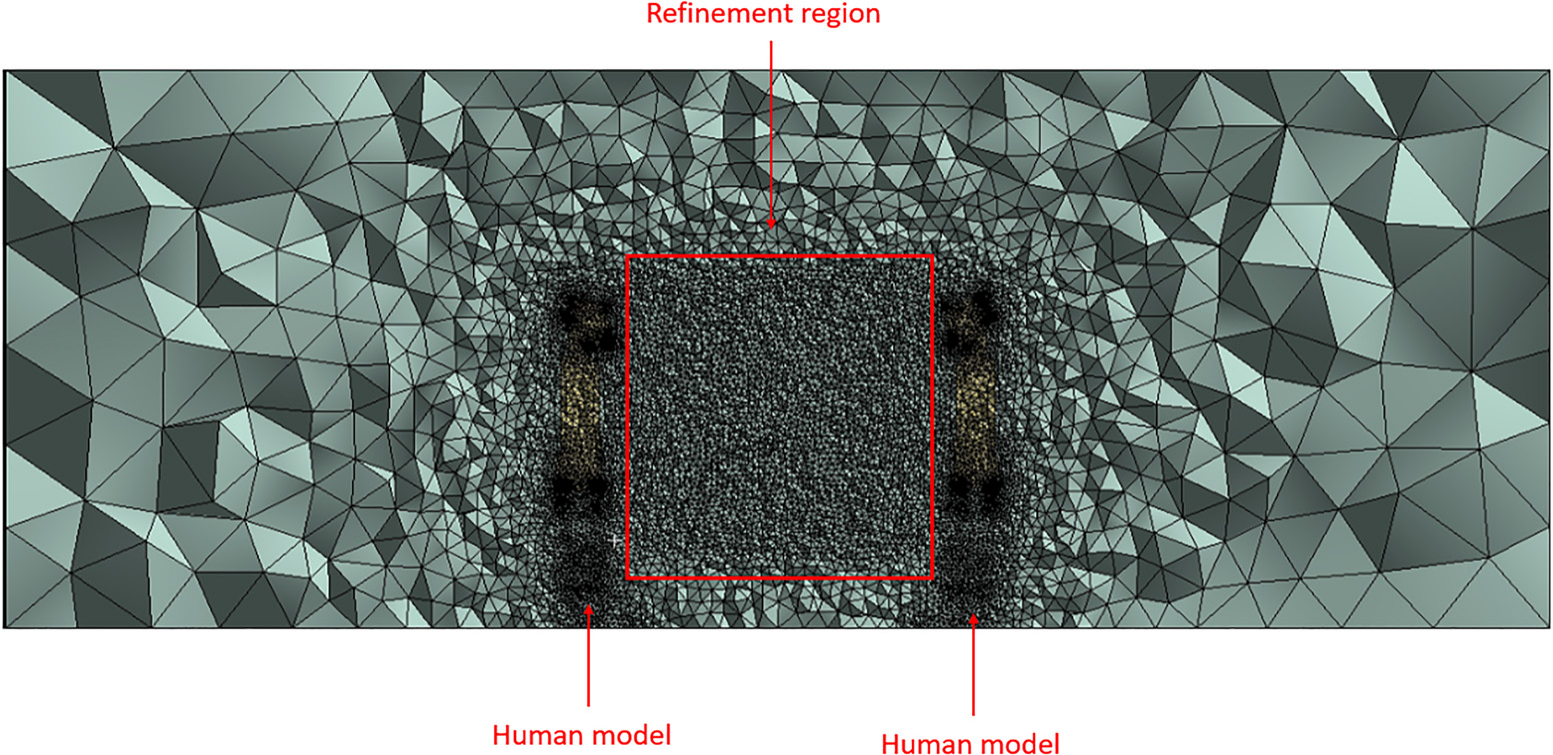
Section view of the full mesh generated.

**FIG. 4.**
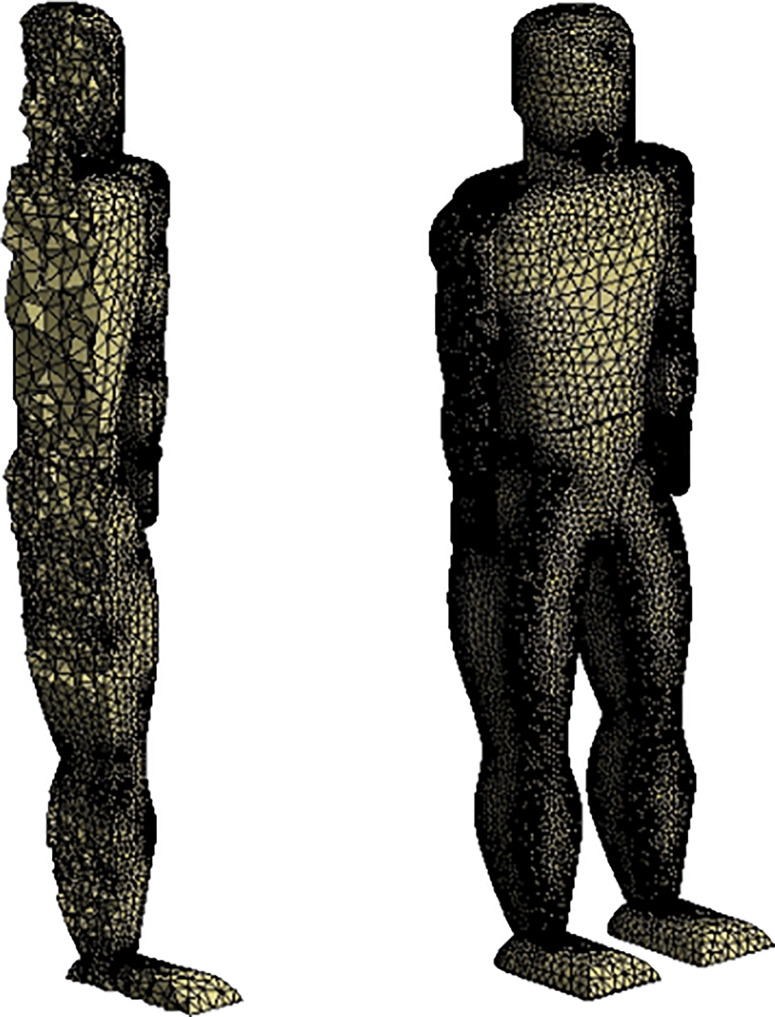
Human model mesh.

The resulting mesh generated is pictured in the following images as an unstructured tetrahedral mesh. Mesh refinement was localized in two regions: the volume where the ejecta is expected to travel through (i.e., the region between the human models) and the human models. Local mesh refinement for the region between the human models was developed based on the advantages discussed by Lanfrit.^30^ Despite the method being quite time-consuming vs other mesh generation methods, the refinement method is capable of producing greater accuracy.^30^ Figure 3 displays the full mesh generated with the refinement region indicated. Figure 4 displays the resulting mesh generated for the human model utilized in the study. The surface mesh for the human model is shown as well as a section view that examines the three-dimensional mesh generated.

#### 2. Mesh independence study

A mesh independence study was conducted to ensure that the simulations performed were not impacted by the mesh size and the solutions are not impacted by the meshes generated. The particle position on the x-axis over a flow time of 1 s was analyzed in each simulation conducted, where conditions are specified in the Model Setup and Solver Settings section. Mesh sizes were altered to decrease by approximately 10% from the previous mesh study. The following Fig. 3 displays the mesh independence study results. Table I lists the mesh sizes and additional information recorded in each study.

**TABLE I.** Mesh sizes used in the study.

Mesh	Mesh size (total elements)	CPU time (s)	Number of CPUs	Memory usage (GB)
Mesh 1	2 501 918	18 646	4	12.20
Mesh 2	2 220 102	15 969	4	11.71
Mesh 3	1 998 092	14 770	4	10.79
Mesh 4	1 733 558	10 864	4	9.37
Mesh 5	1 585 418	10 646	4	8.38
Mesh 6	1 479 787	10 123	4	7.71

**FIG. 5.**
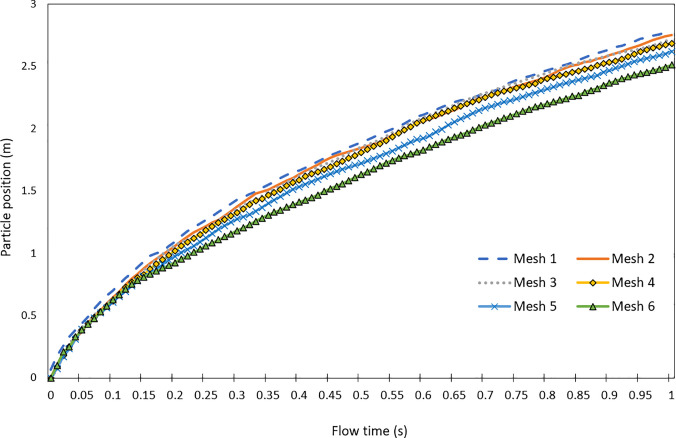
Mesh independence study results.

The results from the mesh independence study display that the difference in particle position results from mesh 1 to mesh 4 is minimal, with approximately a 1% difference in position. This can be identified by viewing Fig. 5. The change from mesh 4 to mesh 5 resulted in an approximate 3% difference in position. The final mesh studied was mesh 5, which had a greater increase in particle position difference with a measurement of 5% change.

The total simulation time from mesh 1 to mesh 6 decreased by 8523 s, as well as the memory usage differed by 4.49 GB. It was found that in most cases the simulation time was impacted by the number of elements present in the study. This can be identified in Table I, where the simulation time decreases as each mesh is studied, with a 15% to 27% decrease in computational time recorded during some studies. The memory usage was also found to be impacted by the number of elements utilized, with the greatest memory usage of 12.20 GB in mesh 1 and the least memory usage of 7.71 GB in the mesh 6. The mesh 4 was chosen as the final mesh to be used in the simulation due to the relatively low simulation time, moderate memory usage, and balance of accuracy and mesh size when compared to the other mesh studies.

### D. Model Setup and Solver Settings

Following the mesh generation are the model setup and solver settings. This aspect of the simulation includes the assignment of boundary conditions and interfacing contacts. A simulation time of one second was used due to the fact that the majority of respiratory particles are transmitted from a coughing or sneezing person in a second of exhalation.^13,17,23^ Also, the 1 s simulation time is very comparable with the other simulation times presented in the literature review. For instance, Busco *et al.*^17^ conducted most simulations up to 0.5 s, and Feng *et al.*^23^ conducted studies with a simulation time of 1 s. The other boundary conditions used in the study are as follows:

1. Multiphase and inlet conditions
2. Discrete phase model injections
3. Porous media conditions

#### 1. Multiphase conditions

As shown in the numerical approach, the Eulerian multiphase model was chosen for its capability of utilizing the dense discrete phase model. The multiphase model was selected with two phases. Phase 1 was selected to be air, and phase 2 was selected to be the injected respiratory droplet particles used to simulate the breathing, sneezing, and coughing effect. As discussed previously, the turbulence model used is k–ω SST. The breathing model utilized inlet conditions of 1.4 m/s, the coughing model used an inlet condition between 1.5 to 28.8 m/s, which was chosen to be 15.5 m/s. Finally, the sneezing model uses an inlet condition between 20 to 50 m/s, chosen to be 35 m/s.^31^

#### 2. Discrete phase model injections

Discrete phase model injections are used to inject a specified mass into the computational domain. This technique is used to simulate the sneezing, coughing, and breathing effects exhibited by the human model. The mass flow rate was specified to be 5.24 × 10^−8^ kg/s with a duration of 0.5 s.^32^

#### 3. Porous media conditions

Porous media conditions were utilized to model the N95 face mask. The face shield did not undergo any porous media changes as it was noted to act more as a solid barrier. The N95 mask was simulated with porosity parameters of 0.88 and a viscous resistance parameter of 1.12 × 10^10^ m^−2^.^33^

#### 4. Wind conditions

The final set of boundary conditions assigned are wind conditions, which were employed based on the Beaufort scale.^34^ The wind conditions of a gentle breeze (4.5 m/s) and moderate breeze (6.7 m/s) were used to identify their impact on particle distance over time.

### E. Simulations Performed

Simulations were conducted using transient conditions. A total flow time of 1 s was chosen to observe the evolution of flow when the respiratory droplets are introduced into the domain. The time step size is 0.01 s with the number of time steps being 100, which is an appropriate value to model the introduction of the particles into the computational domain. This time step size was obtained by the researchers through preliminary studies [i.e., Refs. 18–22]. Sneezing, coughing, and breathing were specified to occur over 0.5 s. Initial simulations conducted were used to identify the effect of physical distancing when no mask was used.

The simulations performed can be split into three categories based on ejection type, with breathing, coughing, and sneezing considered in the simulations. The recommendations made by the World Health Organization and health institutions in the USA and Canada for physical distancing is listed as 1–2 m between individuals.^35–37^ The study employs a recommendation of 2 m for physical distancing to identify the effects of various expulsion methods (breathing, coughing, sneezing). The presence of wind was considered as it can be viewed as having an impact on outdoor physical distancing measures. Thus, wind conditions were incorporated into the model to view the effect that wind would have on the particles, with the wind velocity chosen based on the Beaufort scale.^34^ It should be noted that the N95 face mask was modeled with suboptimal fitting. This is used to display the effects that would occur in the event of incorrect face mask fitting. A review of the literature reveals that the effectiveness of the N95 face masks changes with the fitting of the masks, where improper fitting can result in decreased effectiveness.^38,39^ The simulations performed are listed in Table II.

**TABLE II.** Simulations performed.

Face covering	Ejection type	Wind conditions
No mask	Sneeze	None
No mask	Sneeze	Gentle breeze
No mask	Cough	None
No mask	Cough	Gentle breeze
No mask	Breathing	None
No mask	Breathing	Gentle breeze
No mask	Breathing	Reversed gentle breeze
No mask	Breathing	Moderate breeze
Face shield	Sneeze	None
Face shield	Sneeze	Gentle breeze
N95 mask	Sneeze	None
N95 mask	Sneeze	Gentle breeze

### F. Model Comparison

**FIG. 6.**
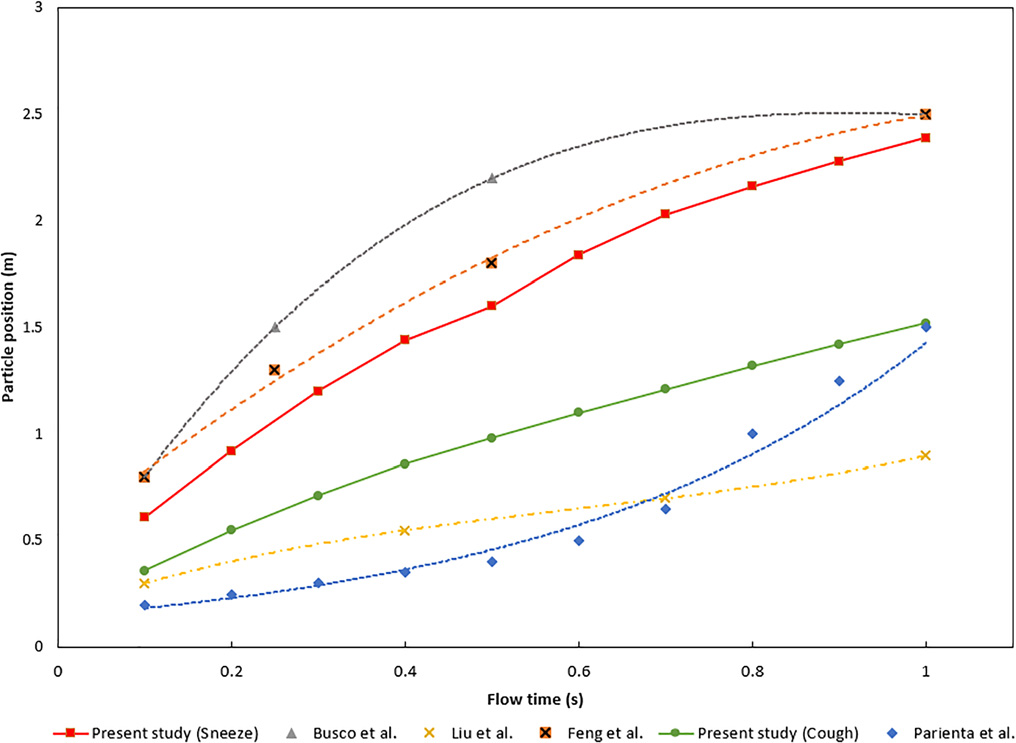
Comparison study results.

The comparison data were obtained through analysis of relevant literature used to study the resulting distances of particles from sneezing and coughing.^17,23,40,41^ The studies were used to identify the distances traveled by the ejected particles and the result is to be compared to the existing model developed in the current study. Busco *et al.*^17^ and Feng *et al.*^23^ analyzed the effect of a sneeze and measured the resulting distances when the ejection occurred. Liu *et al.*^40^ and Parienta *et al.*^41^ analyzed the distance traveled when a cough occurred. The results of the validation study are shown in Fig. 6. It can be observed that the simulation results from this study closely match the beginning and ending positions of the particles in both sneezing and coughing cases, with approximately 10% in those instances. The cough model, however, was found to have differing results from the particle's position before closely matching the study conducted by Parienta *et al.*^41^ near one second of flow time.

## III. RESULTS AND DISCUSSION

In this section, the results are used to examine if the claims of wearing face coverings and maintaining a certain degree of space would be an effective strategy to impede the spread of the respiratory droplets, as well as provide recommendations for a minimum safe distance. All the results discussed in this section have the scenario where an infected human model was facing an uninfected human model of equal stature. The uninfected person, represented by the second human model, provides a visual representation of the offset physical distance provided to the simulation. The model was not excluded from the simulation, as it provides a clear depiction of how respiratory particles can potentially interact with an uninfected person standing directly in front of an infected person.

**FIG 7.**
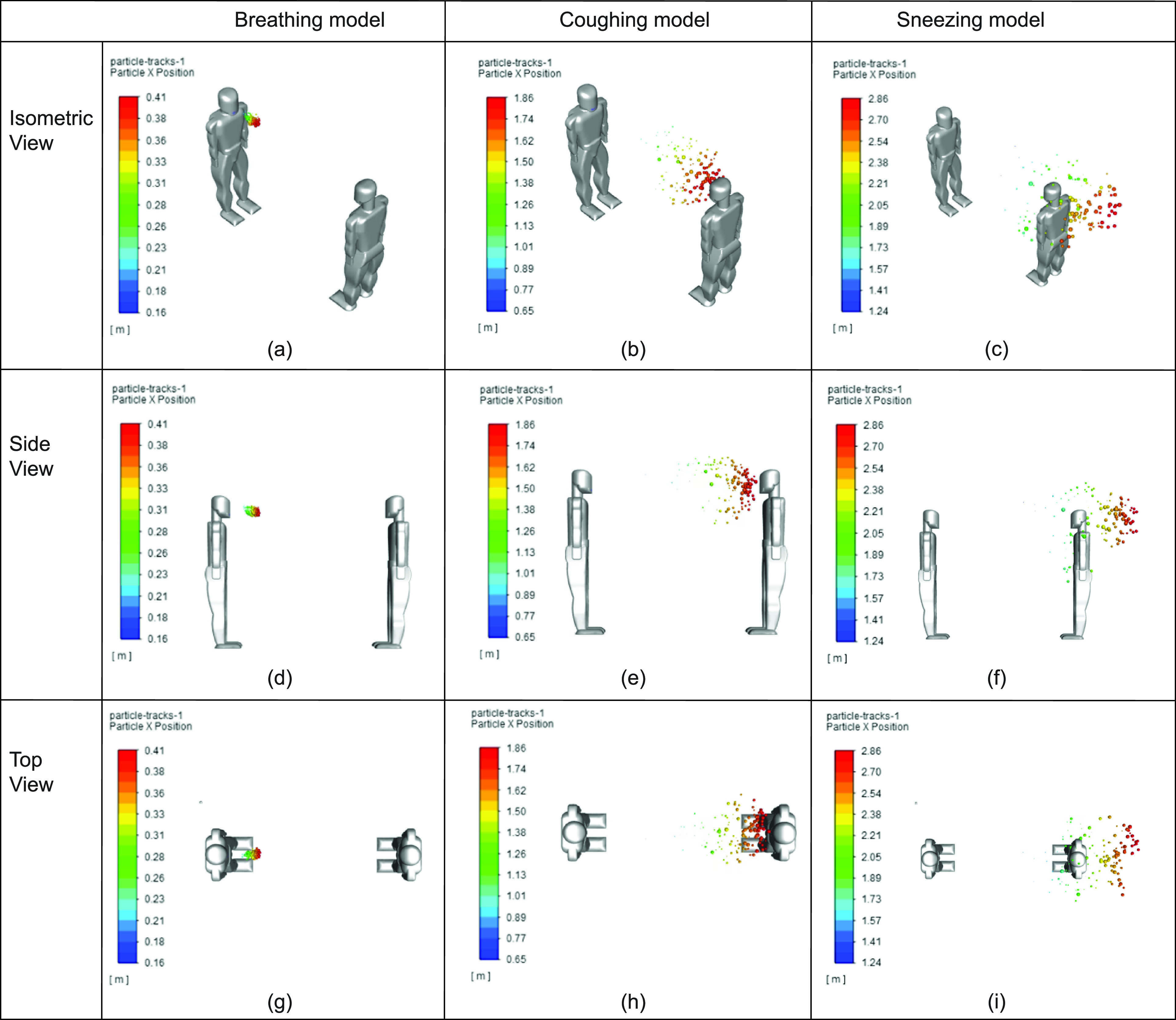
Comparison between breathing, coughing, and sneezing at different views: (a) breathing model (isometric view), (b) coughing model (isometric view), (c) sneezing model (isometric view), (d) breathing model (side view), (e) coughing model (side view), (f) sneezing model (side view), (g) breathing model (top view), (h) coughing model (top view), and (i) sneezing model (top view).

Figure 7 represents how the transmission of respiratory droplets look in breathing, coughing, and sneezing, simulated over 1 s. Within the first second, it was clear that 2 m of space between the human models facing each other is still adequate if the infected person is breathing [i.e., Fig. 7(a)].

However, the coughing in static air simulation reaches substantially closer to the uninfected human model, and the sneezing model [i.e., Fig. 7(c)] even surpasses the location of the uninfected person. This is attributed to the velocity at which the droplets were ejected. Sneezing is released at a 35 m/s, more than double that of coughing, and substantially greater than breathing. Based on this, particles released from sneezing pose a greater risk with regard to the distance they are able to travel.

**FIG. 8.**
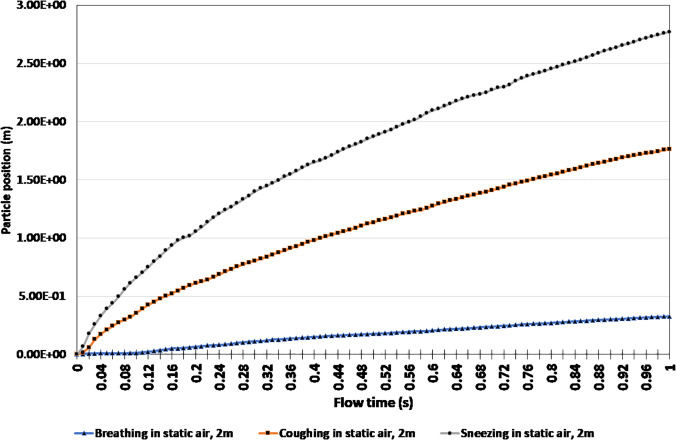
Particle position comparison of breathing, coughing, and sneezing in static air.

Figure 8 displays the change in the position of particles when breathing, coughing, and sneezing over a 1 s time interval in still air. The particle position was used as a measure to determine the furthest distance a respiratory droplet was able to reach within that time frame when two people stand facing each other. As expected, the final particle position of breathing was substantially smaller, only 0.33 m, whereas coughing and sneezing were 1.76 m and 2.77 m, respectively. The 2.77 m particle position exceeds the commonly used physical distancing recommendation of 2 m. Therefore, a new minimum safe distance recommendation of 2.77 m can be made when sneezing in static air.

**FIG 9.**
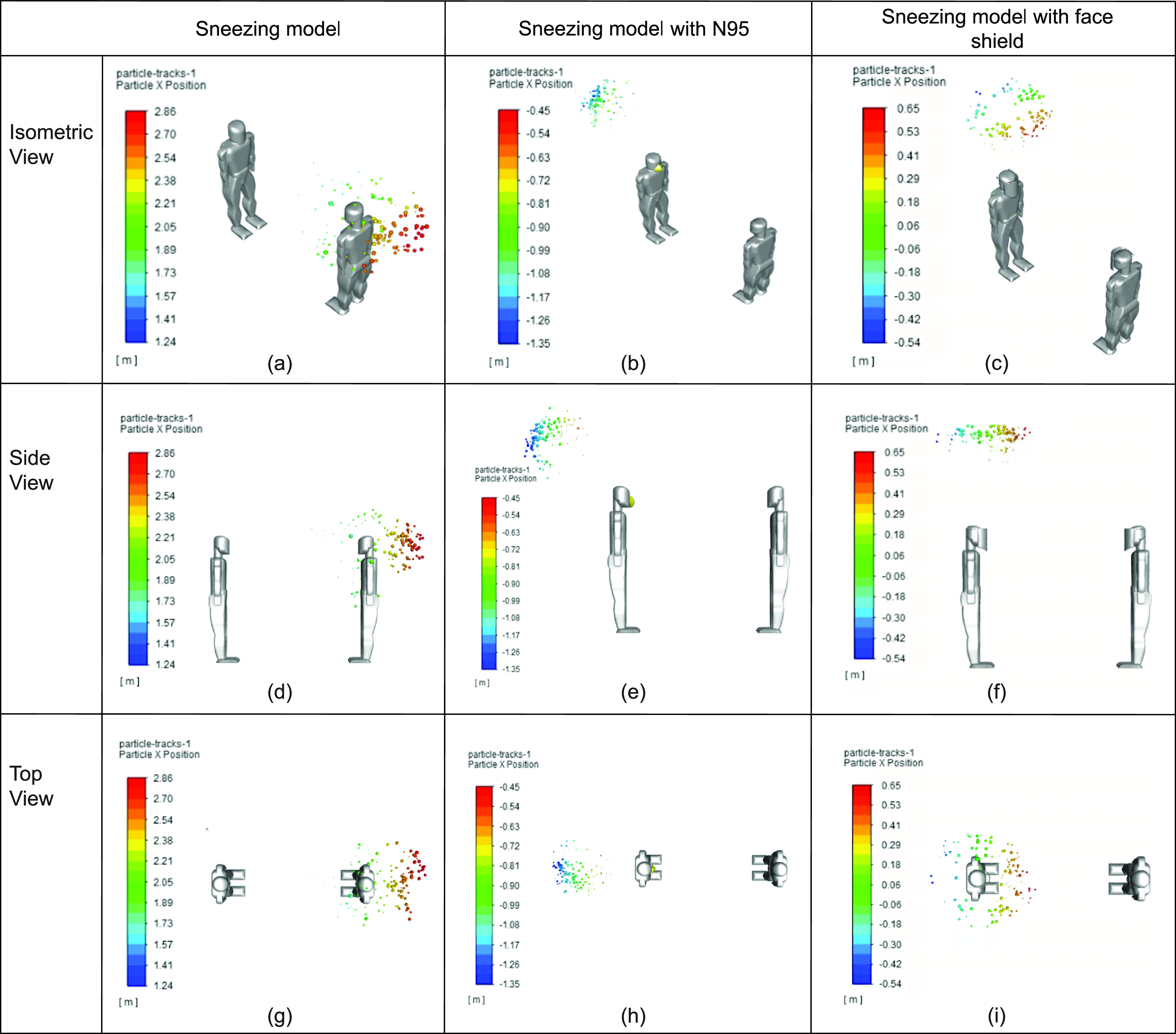
Comparison between sneezing and sneezing while wearing a face-covering at different views: (a) sneezing model (isometric view), (b) sneezing model with N95 (isometric view), (c) sneezing model with face shield (isometric view), (d) sneezing model (side view), (e) sneezing model with N95 (side view), (f) sneezing model (side view), (g) sneezing model (top view), (h) sneezing model with N95 (top view), and (i) sneezing model with face shield (top view).

Given that sneezing displayed the most volatile response from the simulation in regard to the particle position, the impact that preventative measures would have on sneezing was also examined. In order to do this, the previous sneezing scenario, as shown in Figs. 7 and 8, was used to compare sneezing while wearing an N95 mask to sneezing while wearing a face shield. A pictorial representation of these three scenarios is captured in Fig. 9.

As shown in Fig. 9, wearing a face-covering is a highly effective strategy in mitigating the spread of respiratory droplets if the uninfected individual is standing across from the infected individual. In the simulation, the N95 mask was placed several millimeters away from the infected person's face in order to replicate improper fitting of the mask. In the simulation presented, the N95 mask deflected some of the particles which hit it earlier, while others were slowed down [i.e., Figs. 9(b), 9(e), and 9(h)]. This is attributed to the initial parameters set for the N95 mask and its overall shape. Furthermore, the N95 mask was placed at a distance of 3 mm away from the sneezing model to represent improper fitting and was modeled with a porosity of 0.88, therefore inhibiting respiratory particles from seeping through. Also, the model does not take into consideration the particle's ability to stick to the mask. However, the face shield deflected the respiratory droplets more upward. This is likely attributed to the curvature of the face shield, and its close fit around the human model's head during the simulation. The sneeze was without any kind of face-covering served as a reference to show the improvement that wearing a face shield gives.

**FIG. 10.**
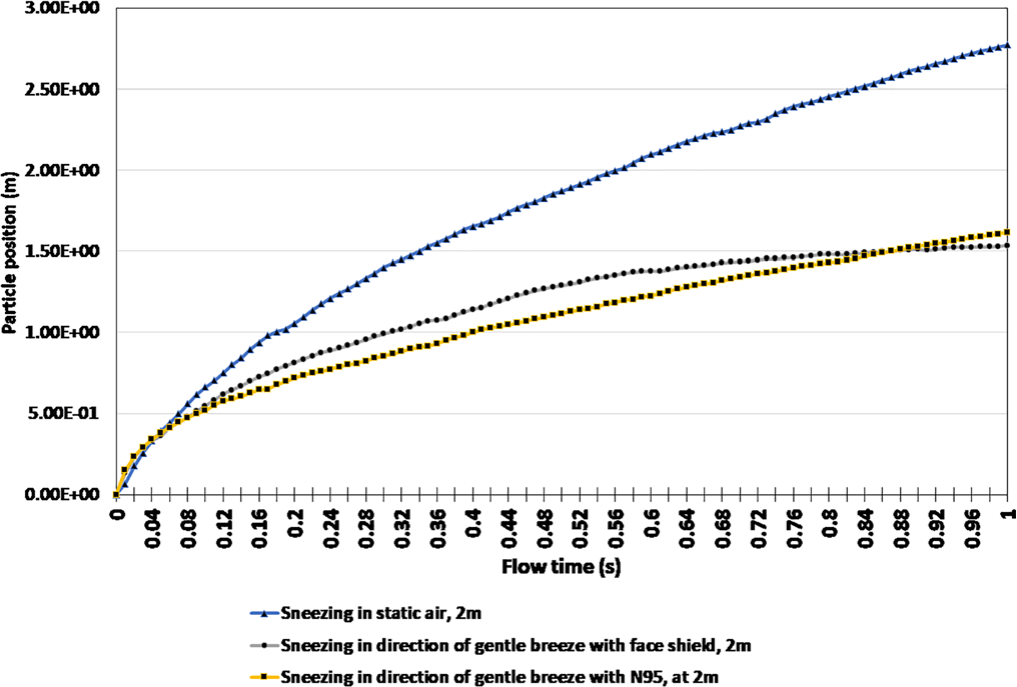
Particle position comparison for sneezing in a gentle breeze.

Figure 10 identifies the impact breezes would have on the position of particles while wearing a face covering. Once again, the results of sneezing without a breeze and without a face covering were used as a baseline to compare the results of sneezing in the direction of a gentle wind while wearing an N95 face covering and a face shield. As expected, the release of respiratory droplets at a high velocity and wind blowing in the direction of the exhalation has the potential to drastically worsen the situation. However, wearing a face covering has the potential to improve the situation. It is evident that in both the N95 and face shield, the simulations achieve a final particle position of 1.62 m and 1.53 m. Therefore, a minimum safe distance recommendation of 1.62 m and 1.53 m can be made in these scenarios. The distance traveled is attributed to respiratory droplets under the influence of the environment after they leave the covering, therefore causing them to be blown forward. Whereas when someone sneezes in static air without the influence of wind, a much larger minimum safe distance recommendation of 2.77 m must be made.

**FIG 11.**
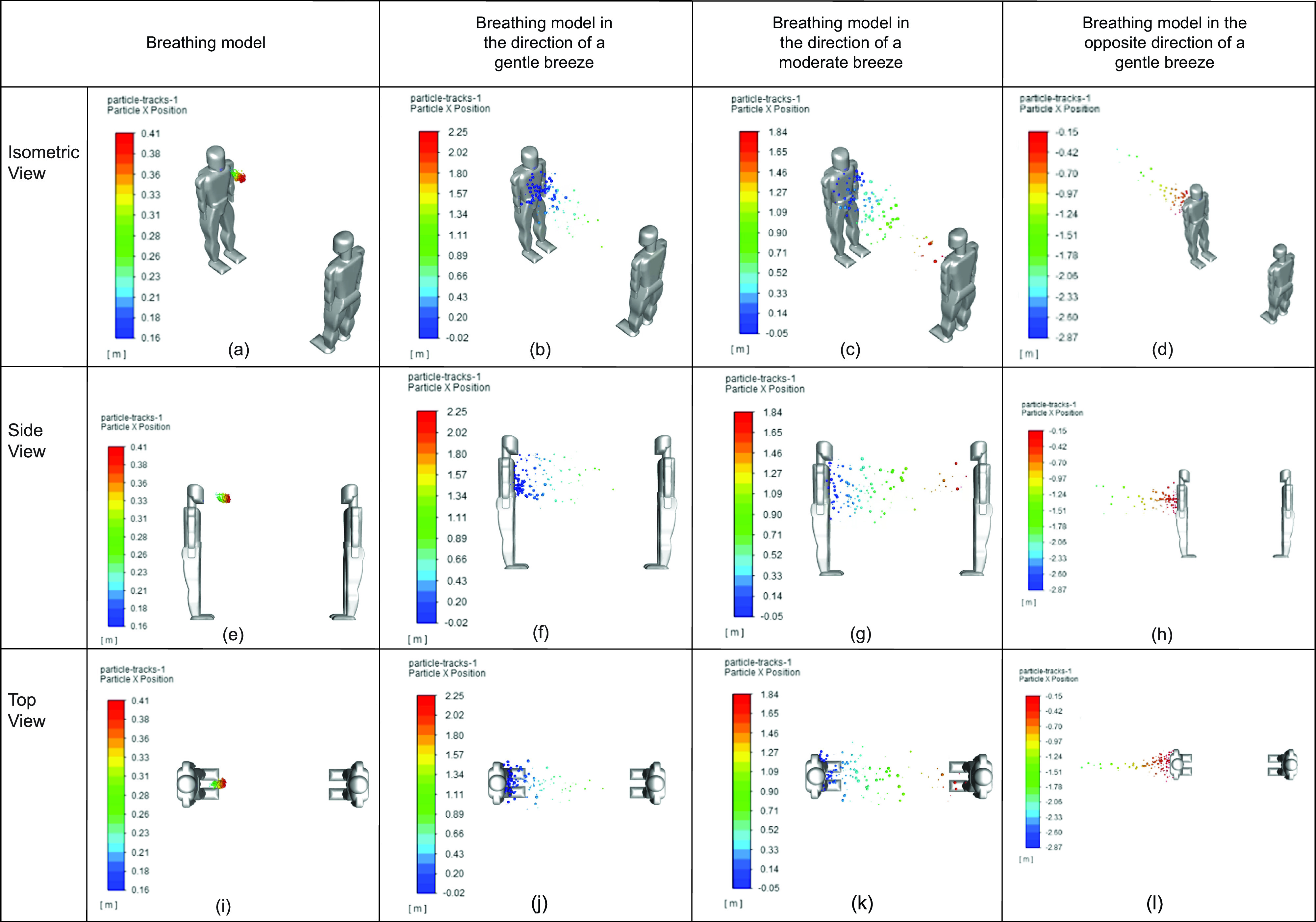
Comparison between breathing in various environmental conditions: (a) breathing model (isometric view), (b) breathing model in the direction of gentle breeze (isometric view), (c) breathing model in the direction of moderate breeze (isometric view), (d) breathing model in the opposite direction of gentle breeze (isometric view), (e) breathing model (side view), (f) breathing model in the direction of gentle breeze (side view), (g) breathing model in the direction of moderate breeze (side view), (h) breathing model in the opposite direction of gentle breeze (side view), (i) breathing model (top view), (j) breathing model in the direction of gentle breeze (top view), (k) breathing model in the direction of moderate breeze (top view), and (l) breathing model in the opposite direction of gentle breeze (top view).

Based on the analysis conducted, it was identified that sneezing produced the most volatile results with and without the influence of wind. This is displayed in the dispersion of particles and the final position of particles. However, it is also acknowledged that these circumstances are somewhat unique and can be considered more extreme. Therefore, breathing analyses in the presence of wind at different velocities and directions were conducted. Figure 11 provides a visual comparison between various scenarios where the infected person is breathing in static air, and in the same direction as a gentle and moderate wind. Finally, the results of the infected person breathing in the opposite direction of a gentle breeze were compared.

As expected, breathing in the same direction of the wind greatly impacts the distance that the respiratory droplets were able to reach. However, the opposite occurred when breathing opposed the direction of a gentle breeze.

**FIG. 12.**
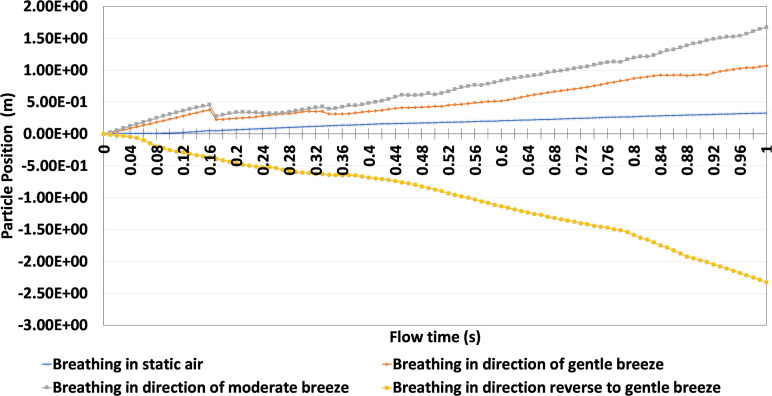
Comparison between sneezing and various face coverings at different views.

The particles' positions from Fig. 11 over time were analyzed in greater detail in Fig. 12. As stated previously, the scenarios presented with greater wind speeds were able to produce further particle positions. This is evident in the fact that breathing in static air produced a final particle position of 0.33 m, whereas breathing in the direction of a moderate wind produced a final particle position 1.67 m. Furthermore, if a gentle breeze blows in the opposite direction of the infected person, the particle position was approximately -2.33 m. The negative particle position implies that the respiratory droplets moved in the opposite direction, and that people behind the person exhaling is primarily at risk as opposed to the person in front.

**FIG. 13.**
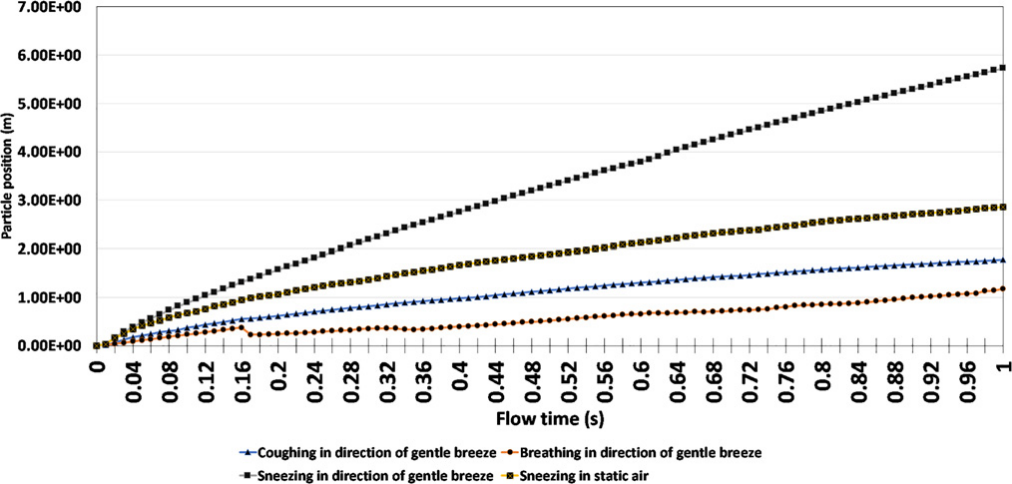
Comparison between sneezing, coughing and breathing with increased physical distance.

Additional analyses were conducted to identify if individuals would still be at risk in more volatile scenarios. To do this, the study compared the results of coughing, breathing, and sneezing in the direction of a gentle breeze with sneezing in static air conditions. Figure 13 provides a graphical representation of the particle's position within the first second of the simulation. Unlike previously conducted studies where the human models were positioned 2 m apart, Fig. 13 increased the position between human models to adjust for environmental conditions and high respiratory particle launch velocities.

Based on the results presented, breathing in the direction of a gentle breeze presents the least risk, given that the particle's final position is 1.18 m at the end of 1 s, whereas sneezing in the direction of a gentle breeze without any face-covering presents a much greater risk, given its maximum particle position of 5.73 m. However, coughing in the direction of a gentle breeze provided a lower level of risk, given its final particle position of 1.78 m. This was likely attributed to the higher velocity of a sneeze compared to a cough. The prevailing notion from the results of Fig. 13 is that physical distance must drastically be increased to adequately protect individuals from the transmission of respiratory droplets, if they are released at a high velocity and in the same direction as a gentle breeze. A minimum safe distance recommendation of 5.73 m can be made if an infected person sneezed in the direction of a gentle breeze.

**FIG 14.**
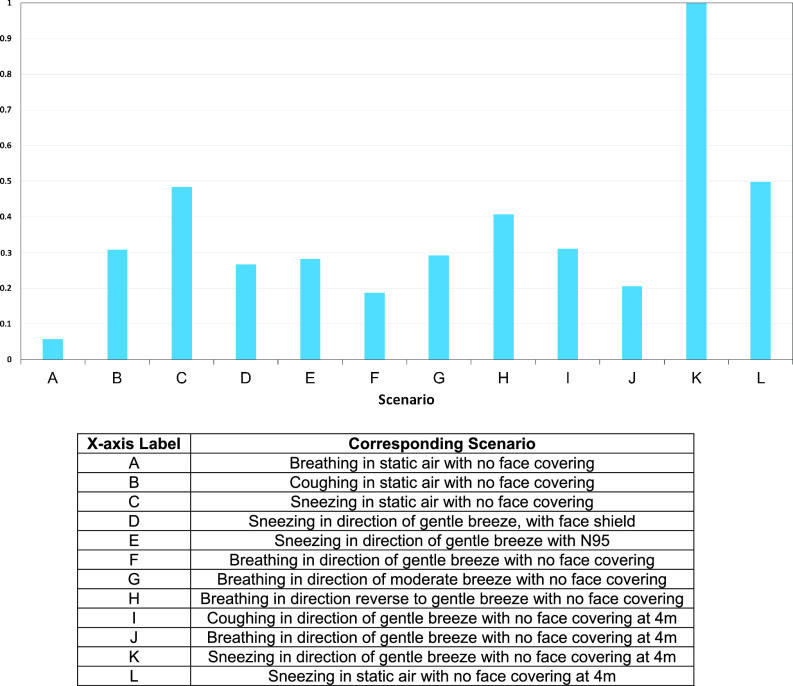
Comparison of COVID-19 risk factors.

An overall score was also assigned to the various simulations presented within the study. This overall score, referred to as the COVID-19 risk factor, falls within the range of 0 to 1, where 1 represents the normalized worst-case scenario of sneezing in the same direction as a gentle breeze and 0 represents breathing, coughing, or sneezing opposite to the direction of a gentle breeze while wearing no face covering(s). This COVID-19 risk factor score of 0 represents relatively no risk to the other person positioned in front of the exhaling person. Figure 14 serves as a representation that compares the COVID-19 risk factor of the various scenarios.

As seen in Fig. 14, sneezing in the direction of a gentle breeze while wearing no face covering produced the worst results. Therefore, all values were normalized relative to this scenario. The results depict that wearing a face-covering can be highly beneficial to mitigate the spread of respiratory droplets. This is illustrated when comparing the COVID-19 risk factor of the subject sneezing in static air without a face covering, with the results of the subject sneezing in the direction of gentle breeze while wearing a face-covering (mask or face shield). Sneezing in the direction of a gentle breeze was considered to be arguably worse environmental conditions; however, in this scenario where subjects wore an N-95 mask and a face shield, COVID-19 risk factors of 0.28 and 0.27 is produced, respectively. However, sneezing in static air with no face covering produced a COVID-19 risk factor of 0.48.

## IV. CONCLUSIONS

In this paper, a series of simulations were conducted to evaluate the transport of respiratory droplets in order to make recommendations on safe physical distancing measures to combat the COVID-19 pandemic. To do this, three scenarios were considered: breathing, coughing, and sneezing. The study also took into consideration the environmental conditions such as wind speed (static, gentle, and moderate) and the direction of the wind (opposite or the same direction of exhalation). All scenarios used two human models standing 2 m and 4 m apart, as a method to verify if the 2 m physical distance recommended by public health officials is sufficient. Two forms of face coverings were also incorporated into the simulations: an N95 mask and a face shield to evaluate if they are indeed effective methods to mitigate or stop the transmission of the virus. Compared to studies reported in the open literature, this paper provides a more comprehensive comparison of various scenarios, including the variation of the environmental wind conditions, face coverings, and distance between human models.

Based on the results discussed, several key conclusions were reached on the transmission of the respiratory droplets with regard to the effectiveness of physical distancing, and mask recommendations. The key conclusions are as follows:

• In the static air simulations, the respiratory droplets produced from breathing were unable to reach the uninfected person. In this scenario, a minimum safe distance recommendation of 0.33 m can be made for breathing in static air. Additionally, a minimum safe distance recommendation from coughing and sneezing in static air was determined to be 1.76 m and 2.77 m, respectively.
• In dynamic air conditions where moderate wind speeds were paired with breathing, which is a more common mode of transmission, the distance that the respiratory particles were able to travel increased significantly. By moving from static wind conditions to moderate wind speeds, the distance traveled by the respiratory droplets increased from 0.33 m to 1.67 m.
• Among the test conducted where face coverings were worn, they provide significant protection to the uninfected person in static and dynamic air conditions, as indicated in Fig. 14 previously.

Based on the key results presented in the study, it was concluded that additional space between people is required and that 2 m may not be sufficient to prevent the transmission of respiration particles, especially if environmental airflow is taken into account or if the exhalation is released at high velocity via sneezing. It was also determined that face-coverings can be a key tool to mitigate the spread of COVID-19; however, they are not capable of providing maximum protection.

## Data Availability

The data that support the findings of this study are available from the corresponding author upon reasonable request.

## NOMENCLATURE

C: matrix in porous media conditions
d: distance from human models (m)
D: matrix in porous media conditions
$D_{\omega }$: cross diffusion term
$D_{\omega }^{+}$: positive portion of cross diffusion term
E: energy (J)
$\vec{F}$: external body forces (N)
${\vec{F}}_{\mathrm{DPM}}$: external forces in dense discrete phase modeling (N)
${\vec{F}}_{\mathrm{lift},q}$: external lift force (N)
${\vec{F}}_{\mathrm{other}}$: other forces in dense discrete phase modeling (N)
${\vec{F}}_{q}$: external body force in multiphase flow (N)
${\vec{F}}_{td,q}$: turbulent dispersion force (N)
${\vec{F}}_{vm,q}$: virtual mass force (N)
${\vec{F}}_{wl,q}$: wall lubrication force (N)
$F_{1}$: blending function
$\vec{g}$: gravitational acceleration (9.81 m s^−2^)
$G_{k}$: production of turbulent kinetic energy (J kg^−1^)
$G_{\omega }$: generation of ω (J kg^−1^)
I: unit tensor
*k*: turbulence kinetic energy (J kg^−1^)
K: interphase exchange coefficient
$\dot{m}$: mass transfer between phases (kg s^−1^)
p: pressure (Pa)
${\vec{R}}_{pq}$: interaction force between phases (N)
S: strain rate magnitude (s^−1^)
$S_{\mathrm{DPM}}$: user-defined function for dense discrete phase modeling
$S_{i}$: porous media momentum source term
$S_{k}$: user-defined source term for k
$S_{m}$: user-defined function for continuity equation
$S_{\mathrm{other}}$: user-defined function for other source terms
$S_{\omega }$: user-defined source term for ω
$\vec{v}$: velocity (m s^−1^)
$\left|v\right|$: magnitude of velocity (m s^−1^)
$V_{q}$: volume fraction of phase q
y: distance to next surface (m)
$Y_{k}$: dissipation due to turbulence for k (J kg^−1^)
$Y_{\omega }$: dissipation due to turbulence for ω (J kg^−1^)

**Symbols**
α: phase volume fraction
$\alpha^{*}$: turbulent viscosity damping coefficient
$\Gamma_{k}$: effective diffusivity of k (m^2^ s^−1^)
$\Gamma_{\omega }$: effective diffusivity of ω (m^2^ s^−1^)
ϵ: turbulence dissipation rate (J kg^−1^ s^−1^)
$\lambda_{q}$: bulk velocity for phase q (m s^−1^)
μ: molecular viscosity (m^2^ s^−1^)
$\mu_{q}$: shear velocity for phase q (m s^−1^)
$\mu_{T}$: turbulent viscosity (m^2^ s^−1^)
ρ: density (kg m^−3^)
$\sigma_{k}$: turbulent Prandtl number for k
$\sigma_{\omega }$: turbulent Prandtl number for ω
$\overset{̿}{\tau }$: stress tensor (Pa)
${\overset{̿}{\tau }}_{q}$: phase stress–strain tensor (Pa)

**Acronyms**
CDC: Centers for Disease Control and Prevention
CFD: computational fluid dynamics
MERS: Middle East respiratory syndrome
SARS: severe acute respiratory syndrome
SST: shear stress transport
